# Gender Differences in Problematic Alcohol Consumption in University Professors

**DOI:** 10.3390/ijerph14091069

**Published:** 2017-09-15

**Authors:** Pablo Ruisoto, Silvia L. Vaca, José J. López-Goñi, Raúl Cacho, Iván Fernández-Suárez

**Affiliations:** 1 Department of Basic Psychology, Psychobiology and Methodology of Behavioral Sciences, University of Salamanca, 37008 Salamanca, Spain; 2 Department of Psychology, European University of Madrid, 28670 Villaviciosa de Odon, Spain; 3 Department of Psychology, Universidad Técnica Particular de Loja, 11-01-608 Loja, Ecuador; slvaca@utpl.edu.ec; 4 Department of Psychology and Pedagogy, Public University of Navarra, 31006 Pamplona, Spain; josejavier.lopez@unavarra.es (J.J.L.-G.); raul.cacho@unavarra.es (R.C.); 5 Department of Behavioral Sciences, International University of La Rioja, 26004 Logroño, Spain; ivan.fernandez@unir.net

**Keywords:** alcohol, gender, university, professors, job satisfaction

## Abstract

The role of job satisfaction and other psychosocial variables in problematic alcohol consumption within professional settings remains understudied. The aim of this study is to assess the level of problematic alcohol consumption among male and female university professors and associated psychosocial variables. A total of 360 professors (183 men and 177 women) of a large private university in Ecuador were surveyed using standardized instruments for the following psychosocial measures: alcohol consumption, job satisfaction, psychological stress, psychological flexibility, social support and resilience. Problematic alcohol consumption was found in 13.1% of participants, although this was significantly higher (χ^2^ = 15.6; d.f. = 2, *p* < 0.001) in men (19.1%) than women (6.8%). Problematic alcohol consumption was reported in men with higher perceived stress and job satisfaction. However, 83.3% of women with problematic alcohol use reported lower job satisfaction and higher psychological inflexibility. Results suggest that job satisfaction itself did not prevent problematic alcohol consumption in men; stress was associated with problematic consumption in men and psychological inflexibility in women. Findings from this study support the need to assess aspects of alcohol consumption and problematic behavior differently among men and women. Intervention strategies aimed at preventing or reducing problematic alcohol consumption in university professors must be different for men and women.

## 1. Introduction

Problematic drinking is currently one of the major public health challenges in the Western world due to its high prevalence [1,2], which is negatively associated with poor mental health and other major harmful major consequences, including domestic violence [3], increase of absenteeism and reduction of labor productivity [4,5]. Problematic alcohol consumption varies across professions, although most studies have focused mainly on unskilled labor: metro employees (7%) [6], transportation sector (8.4%) [7], sea-related jobs (fishermen, sailors, divers) (12%) [8] textile factories workers in Mexico (25%) [9]. Rates of problematic alcohol consumption also vary over time within the same profession. For example, from 1980 to 2005, problematic alcohol consumption in the US military grew from 15 to 20% [10]. The current prevalence of problematic alcohol consumption in skilled professions such as university professors remains unknown. 

From a psychosocial perspective, working conditions are considered social determinants of health and health-related behavior, such as alcohol consumption, considered an unhealthy coping strategy in stressful situations [11,12] that may result in burnout or depression [12,13], aggravating alcohol consumption [13]. In this context, the nature of the work environment plays a key role in alcohol consumption [14].

The relationship between job satisfaction and worker health has been extensively studied. However, the extent of the relationships varies widely [4,13,14]. 

Lack of psychological resources such as self-regulation has been recently reformulated as psychological inflexibility [15], or the ability to recover from difficult situations, or resilience [16,17]. In addition, loneliness [18] has also been suggested to increase the risk of alcohol consumption, negatively affecting worker health and productivity in the workplace [19].

It has been argued that biological, psychological and social differences for males and females may result in sex-specific patterns of occupational health problems that should be further explored [20,21].

Finally, problematic alcohol consumption in low- and middle-income countries [14], especially in highly skilled occupations, remains understudied and the associated psychosocial variables need further research [5]. 

In line with the recommendations of the “Global strategy to reduce the harmful use of alcohol” [14] and “Gender, health and work” published by the World Health Organization, the aim of this study is twofold: (1) firstly, to assess the level of problematic alcohol consumption among male and female university professors; and (2) secondly, to target specific high-risk groups in this population in a middle-income country.

## 2. Materials and Methods

### 2.1. Participants

Table 1 shows the sociodemographic characteristics of the sample. 

### 2.2. Measures

The following sociodemographic variables were included in the questionnaire: sex, age, marital status and professional category as professor.

*Alcohol Use Disorders Identification Test (AUDIT*, Self-report version) [22]. Consists of 10 items to assess problematic alcohol consumption. Subjects respond by indicating the frequency of alcohol consumption and/or symptoms related to problematic drinking, 0 = “never”, 4 = “4 or more times a week”. Scores range from 0 to 40. Higher scores indicate higher risk of problematic alcohol consumption. AUDIT is the most commonly used test to measure alcohol consumption Cronbach’s alpha coefficient for internal consistency reliability was α = 0.81 for males α = 0.70 and females. 

*Working Environment Scale (WES-10)* [23]. This consists of a 10-item questionnaire for assessing the an overall degree of satisfaction with the perceived climate at work in terms of self-realization, workload, conflict and nervousness. Participants respond to a 5-point Likert-type scale, from 0 = “not at all” to 4 = “to a very large extent”. The higher the score, the higher the degree of job satisfaction. Cronbach’s alpha coefficient for internal consistency reliability was α = 0.71 for males α = 0.74 and females. 

*Perceived Stress Scale (PSS-14)* [24]. This consists of 14 items for assessing the degree to which people perceive lack of control in their daily lives. Participants respond to a 5-point Likert-type scale ranging from 0 (never) to 4 (very often). Scores range from 0 to 56 points. Higher scores indicate higher levels of stress. It has good psychometric properties and correlates with cortisol measurements in blood and saliva. Cronbach’s alpha coefficient for internal consistency reliability was α = 0.82 for males and α = 0.83 for females.

*Avoidance and Action Questionnaire (AAQ-7)* [25]. This is the most widely used general measure of psychological inflexibility, defined as rigidity in the handling of emotions or unpleasant internal events. It consists of 7 items and participants respond to a 7-point Likert-type scale, from 1 = “never” to 7 = “always”. Scores range from 7 to 49. Higher scores indicate tendency to act under the need to control or avoid aversive thoughts, memories or feelings. Cronbach’s alpha coefficient for internal consistency reliability was α = 0.93 for males and α = 0.95 females.

*UCLA Loneliness Scale Revised-Short* [26]. This consists of a brief 3-item scale evaluating the subjective feeling of loneliness, understood as the perception of less social support being available than desired. Participants respond based on their agreement with previous statements, 1 = “hardly ever”, 2 = “sometimes”, and 3 = “often”. Scores range from 0 to 9. Higher scores indicate greater feeling of loneliness or lack of social support. Cronbach’s alpha coefficient for internal consistency reliability was α = 0.76 for males and α = 0.84 for females.

*Brief Resilience Scale* [27]. This is a 6-item scale that assesses resilience as a coping style characterized by the ability to resist disease, and to recover from stressful situations. Participants respond to a 5-point Likert-type scale, where 1 = “strongly disagree”, and 5 = “strongly agree”. The higher the score, the greater the ability to overcome stress. Cronbach’s alpha coefficient for internal consistency reliability was α = 0.82 for males and α = 0.83 for females.

### 2.3. Design and Procedure

A descriptive cross-sectional study was conducted. Approval was obtained by the local ethics committee of the university, and all participants completed an informed consent in accordance with the principles set out in the Helsinki Declaration (UTPL_CB_2014_001). All professors (*n* = 454) at one of the largest private universities in the south of Ecuador received an invitation via email to anonymously participate in the study. Participants were invited to complete a computerized survey during the first three weeks of November 2015, and received a brief report with their results (without revealing personal information) to foster commitment and honesty in the answers. The participation rate was 79.3% (*n* = 360 teachers). A total of 94 participants (20.7% of the sample) were excluded from the study because they did not complete the survey by the end of the 3-week assessment period or the questionnaire was completed faster than required for a comprehensive reading of the items. Participation was confidential, fully anonymous, and a brief summary of individual scores was freely provided after completion of the survey to encourage honest answers and a higher response rate.

### 2.4. Statistical Analysis

Descriptive analyses were performed for all variables. Then χ^2^ analysis or Student’s *t*-test were used to compare independent groups analyzing differences between male and female professors. The sample was divided into three groups according to the scores obtained in the AUDIT: (1) those who did not consume alcohol (Group a); (2) those who presented non-problematic alcohol consumption (AUDIT < 7) (Group b); and (3) those who reported problematic alcohol consumption (including people at risk, harmful or dependent in AUDIT terminology) (scores > 7) (Group c) [22]. Comparisons between these 3 groups were conducted applying χ^2^ test or the analysis of variance, depending on the nature of the variables, and considering adjusted significance levels of *p <* 0.05, 0.01 and 0.001.

The descriptive analysis was based on 3 groups (non-consumers, non-problematic consumers and problematic alcohol consumers). Chi-squared Automatic Interaction Detection (CHAID) multivariate analysis was conducted separately for males and females, establishing cut-off points for the different scales between problematic alcohol consumers and the rest (non-consumers or moderate/non-problematic consumers). There were two reasons for this; firstly, the small size of the non-consumer group (*n* = 24); and secondly, the main focus of this study is to identify risk groups for problematic alcohol consumption. Moderate consumers have been reported to be less informative or relevant for work-related health [28]. 

The CHAID technique evaluates the discriminant capacity of a nominal variable (in this case assignment to one of the two groups) through the significance of χ^2^ [29], and has been used with good results in general [30] and clinical [31] populations. In addition, the Odds Ratio for problematic alcohol consumption was calculated in each of the subgroups or nodes found in the discriminant multivariate analysis versus the rest of the sample. All statistical analyses were performed using the SPSS statistical package 15.0 (SPSS Inc., Chicago, IL, USA).

## 3. Results

### 3.1. Prevalence of Alcohol Consumption Among College Professors

Alcohol consumption was significantly higher in male than female professors (χ^2^ = 15.6, d.f. = 2, *p* < 0.001). Considering the categories of alcohol consumption established in the AUDIT [23]: 18.1% (*n* = 65) of professors reported no alcohol consumption, 13.1% (*n* = 24) of male professors and 23.2% (*n* = 41) of female professors; 68.9% (*n* = 248) reported non-problematic alcohol consumption, 67.8% (*n* = 124) of male professors and 70.1% (*n* = 124) of female professors; and 13.1% (*n* = 47) reported problematic of alcohol consumption levels, 19.1% (*n* = 35) of male professors and 6.8% (*n* = 12) of female professors. 

Table 2 and Table 3 show the psychosocial profile of male and female professors based on their alcohol consumption level. Men with problematic alcohol consumption reported significantly higher scores in loneliness, more psychological inflexibility, psychological stress and lower resilience (Table 2). Women with problematic alcohol consumption reported significantly higher psychological inflexibility and higher psychological stress (Table 3).

### 3.2. Problematic Alcohol Consumption in Male College Professors

**Figure 1:**
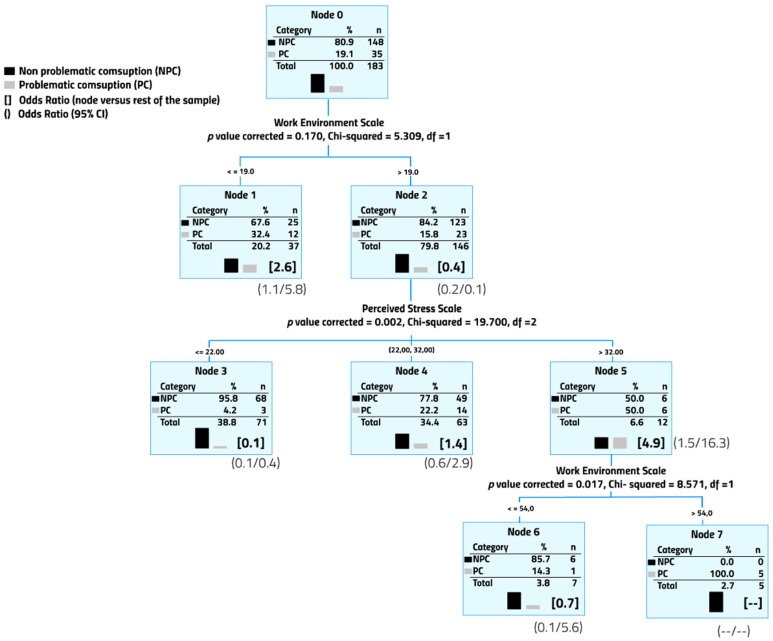
Male professor subsamples identified based on the degree of problematic alcohol consumption. All variables considered in the study were included in the CHAID analysis.

From a multivariate perspective, male professors were divided into 7 subgroups (or nodes) based on the presence or absence of problematic alcohol consumption (Figure 1). The final model included 2 of the 6 scales considered in the study. All male professors who scored higher than 23 on the job satisfaction scale and 32 on the stress scale reported problematic alcohol consumption (Node 7). Conversely, professors who scored higher than 19 in job satisfaction and under 23 in stress reported the lowest percentage of problematic alcohol consumption (Node 3, Odds Ratio = 0.1).

### 3.3. Problematic Alcohol Consumption in Female College Professors

**Figure 2:**
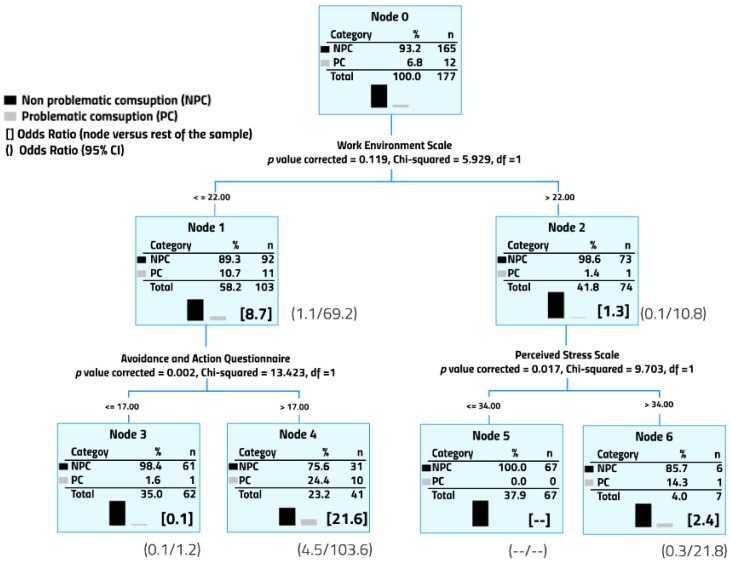
Female professor subsamples identified based on the degree of problematic alcohol consumption. All variables considered in the study were included in the CHAID analysis.

Female college professors were divided into 6 subgroups (Figure 2). The final model included 3 of the 6 scales considered in the study. 10 of the 12 reported cases of problematic alcohol consumption were found in a single subgroup whose score was less than 23 in job satisfation and higher than 17 in psychological inflexibility (Node 4, Odds Ratio = 21.6). On the other hand, absence of problematic alcohol consumption was defined by scores higher than 22 in job satisfaction and lower than 35 in stress (Node 5). 

Finally, Table 4 presents the Odds Ratio (previously reported in Figure 1 and Figure 2) associated with problematic alcohol consumption in the different nodes from higher to lower. On the one hand, the highest alcohol consumption scores in male professors were concentrated in node 7 (all professors included here had problematic alcohol consumption), node 5 and node 1; female professors with the highest scores were concentrated in nodes 4 and 1. On the other hand, male professors with the lowest odds ratio of problematic alcohol consumption were in node 2; none of the female professors in node 5 reported problematic alcohol consumption.

## 4. Discussion

This study is the first, to our knowledge, that (1) explores the current rate of problematic alcohol consumption in university professors in a middle-income country, and (2) targets specific high-risk problematic alcohol consumption groups.

The rate of problematic alcohol consumption among this population was 13.1%, higher than the rate previously found in low-skilled jobs in high income countries, such as transport workers [7] or metro workers [6] in Spain, was similar to the rate found in seafarers in Spain [8], and lower than the rate reported by the military in the USA [10], or for low-skilled jobs in low- or middle-income countries [9]. These findings suggest that complexity or skill-level of occupations and national income level provide environmental clues for predicting problematic alcohol consumption rates. The high rate of problematic alcohol consumption found among university professors represents a source of concern in environments such as universities. Alcohol leads to increases in health-related complications, absenteeism and lower quality and quantity of work due to poor decision-making, as well as a greater risk of getting fired [5,14]. Professors (both males and females) with higher stress, psychological inflexibility and lower resilience reported higher levels of problematic alcohol consumption. These results are consistent with the literature that agrees on the negative impact these aspects have on health [32,33,34,35]. Considering that most professors in our sample were in their late 30 s, there is a need to prevent future alcohol-related health complications by promoting more adaptive coping work strategies.

This study found that male university professors reported a rate of problematic alcohol consumption three times higher than female professors. This ratio 3:1 was higher than the ratio 2:1, males:females, reported in previous studies [36]. Differences in the complexity of the occupations or income-level of the country might partially account for this difference. 

The primary gender differences were found in two psychosocial variables: loneliness and job satisfaction. First, loneliness was associated with significantly higher rates of problematic alcohol consumption only in male professors. This pattern has only been previously reported in a sample of Ecuadorian university students [37]. In general, most studies have focused on exploring loneliness across a lifetime, in samples from high-income countries [38,39]. Second, lower job satisfaction was associated to with significantly higher rates of problematic alcohol consumption only in female professors. Previous studies have reported a positive relationship between job dissatisfaction and problematic alcohol consumption [18,40]. However, in our study, job satisfaction itself did not prevent problematic alcohol consumption in male professors. In our view this result emphasizes that female professors who were exposed to a more adverse work environment, or reported more job dissatisfaction, may develop a typical male-like stress coping style such as alcohol consumption [41]. This finding is important, because women usually suffer discrimination, mobbing and harassment more often than men, especially when they have non-traditional, complex or highly skilled occupations [42]. This finding may benefit future programs aimed at improving work health in female professors.

One of the most striking results to emerge from this study is the revelation of different high-risk problematic alcohol consumption groups for male and female professors, based on a more detailed analysis of the results from the multivariate CHAID analysis. 

On the one hand, the highest-risk of problematic alcohol consumption for male professors was best predicted by using cut-off points in two scales: perceived stress and job satisfaction. Male professors with higher stress reported 5 times more risk of problematic alcohol consumption than those with lower stress (node 5, Figure 1). This finding is consistent with literature on job stress in the work environment and alcohol consumption [41]. Interestingly, a subgroup of male professors with higher stress, who also scored higher in job satisfaction, reported the highest risk of problematic alcohol consumption: 100% (node 7, Figure 1). This result might look contradictory at first glance; however, it could be speculated that alcohol consumption in male professors is associated with two scenarios: alcohol consumption as a coping behavior for stressful situations [18,41], and alcohol consumption as a behavior linked to success or satisfaction in competitive work environments such as universities. As a result, the highest risk factor for alcohol consumption in male professors would be expected when both high stress and job satisfaction are present. Alcohol can be part of a balanced lifestyle when consumed moderately and responsibly; however, in this subgroup, the rate of problematic alcohol consumption may be seriously harmful to the user and to others [42]. Interventions to reduce problematic alcohol consumption in male university professors should target those with higher stress, paying special attention to the ones who report higher job satisfaction. 

Another high-risk subgroup was found among male professors with low stress, defined by low job satisfaction. This subgroup reported 2.6 times more risk of problematic alcohol consumption than the rest (node 1, Figure 1). The subgroup with the lowest risk of problematic alcohol consumption was established firstly by low perception of stress, then by presence of high job satisfaction (node 3, Figure 1). Both results were consistent with the previous literature [40,41].

On the other hand, the highest risk of problematic alcohol consumption for female professors was best defined by cut-off points in two scales: psychological inflexibility and job satisfaction. Female professors who scored lower in job satisfaction and higher in psychological inflexibility reported a 21.6 times higher rate of problematic alcohol consumption than female professors with higher job satisfaction and lower psychological inflexibility (node 4, Figure 2). This finding is consistent with previous studies that highlight the negative health implications of psychological inflexibility, understood as the lack of ability to adapt to fluctuating situational demands and balancing competing needs in life [34,36], as well as job dissatisfaction [18,40,41,42,43]. In addition, although the rate of problematic alcohol consumption in female professors was relatively low (6%), the most significant result in this study was that the highest-risk subgroup, established by the combination of these two variables, accounted for 83% of the total cases with problematic alcohol consumption in this sample. Interventions aiming to reduce problematic alcohol consumption in university female should target both variables, reducing psychological inflexibility by creating nurturing environments that help cope with change [44] and increase job satisfaction. Several approaches have proven effective: improving the effort-reward balance [45], reducing job insecurity [46], and enhancing social support perception [47].

In summary, this study broadens our knowledge of problematic alcohol consumption in highly complex or skilled work environments and in the global context of middle income countries. This is important for three main reasons; first, because most current evidence on problematic alcohol consumption comes from high-income countries, but alcohol consumption affects the poor disproportionately; therefore, lower- and middle-income countries are expected to report higher levels, as we have found in this study, and more severe consequences [1,48]; second, because the assessment and management of health risks is considered a priority at the workplace [49,50], and this study provides useful information to guide gender-specific interventions aimed at reducing problematic alcohol consumption in the working environment [51,52]; third, because the gender-related differences and high-risk groups for problematic alcohol consumption found in this study may reflect social gender inequalities or differences in the role alcohol consumption plays in the context of middle-income countries. Since problematic alcohol consumption has consistently been regarded as a key risk factor for violence towards women, which is a widespread social problem in Ecuador [53], results from this study may improve interventions designed to reduce harmful alcohol consumption, therefore preventing or reducing violence towards women in middle-income countries.

Finally, these results should be generalized with caution, since this is a cross-sectional study based on self-reporting in a single large university in Ecuador. Further research on gender differences in problematic alcohol consumption is encouraged, specifically in understudied complex high-skilled occupations in middle-income countries.

## 5. Conclusions

The rate of problematic alcohol consumption in university professors was 13.3%, with a ratio 3:1 among males and females, a similar rate to non-skilled workers in high-income countries. 

The highest risk group for problematic alcohol consumption was defined by higher perceived stress and not by job satisfaction in male professors, and by psychological inflexibility and low job satisfaction in female professors.

Interventions aiming to promote a healthy work environment and reduce problematic alcohol consumption should consider gender differences in harmful consumption and high-risk groups.

## Figures and Tables

**Table 1:** Sociodemographic characteristics of the sample.

	Total M ± SD (*n* = 360)	Male Professors M ± SD (*n* = 183)	Female Professors M ± SD (*n* = 177)	t (d.f.)	*p*
Age	38.3 ± 8.8	39.3 ± 8.7	37.3 ± 8.8	2.1 (358)	0.036
Experience (years)	7.6 ± 6.9	7.5 ± 7.7	7.7 ± 6.0	0.3 (358)	0.757
	*% (n)*	*% (n)*	*% (n)*	χ^2^ (d.f.)	*p*
Full-time professor	38.3 (138)	33.9 (62)	42.9 (76)		
Full-time assistant professor	39.7 (143)	40.4 (74)	39.0 (69)	4.3 (2)	0.114
Part-time professor	21.9 (79)	25.7 (47)	18.1 (32)		
Single	28.9 (104)	23.5 (43)	34.5 (61)		
Married	63.3 (228)	71.0 (130)	55.4 (98)	9.9 (3)	0.019
Divorced	7.2 (26)	4.9 (9)	9.0 (17)		
Widow	0.6 (2)	0.5 (1)	0.6 (1)		

Note: Total age ranged from 23 to 59 years old. A total of 90% of professors had less than 7 years of experience.

**Table 2:** Psychosocial variables associated with the alcohol consumption level (AUDIT) in male professors (*n* = 183).

	No Alcohol Consumption M ± SD (*n* = 24)	Non-Problematic-Consumption M ± SD (*n* = 124)	Problematic-Alcohol-Consumption M ± SD (*n* = 35)	*F*	*Post-Hoc*
Loneliness Scale	5.26 ± 2.14	5.68 ± 2.07	6.44 ± 2.15	2.9	c > a **, b *
Brief Resilience Scale	23.67 ± 3.94	22.59 ± 4.51	20.18 ± 5.37	5.6 **	(a, b) ** > c
Avoidance and Action Questionnaire (psychological inflexibility)	12.63 ± 6.37	14.82 ± 7.05	20.87 ± 9.89	12.4 ***	c > (a, b) ***
Job satisfaction (WES-10)	23.04 ± 3.66	22.07 ± 4.06	21.67 ± 3.72	1.0	
Perceived Stress Scale	20.22 ± 8.19	22.54 ± 6.51	26.44 ± 6.92	7.6 **	c > a ***, b **

* *p* < 0.05; ** *p* < 0.01; *** *p* < 0.001. Group a = No alcohol consumption; Group b = Non-problematic-consumption; Group c = Problematic-alcohol-consumption.

**Table 3:** Psychosocial variables associated with the alcohol consumption level (AUDIT) in female professors (*n* = 177).

	No Alcohol Consumption M ± SD (*n* = 41)	Non-Problematic-Consumption M ± SD (*n* = 124)	Problematic-Alcohol-Consumption M ± SD (*n* = 12)	*F*	*Post-Hoc*
Loneliness Scale	5.75 ± 2.59	6.05 ± 2.11	7.67 ± 2.50		
Brief Resilience Scale	20.96 ± 6.00	21.42 ± 4.34	18.8 ± 4.92	2.1	b > c *
Avoidance and Action Questionnaire (Psychological inflexibility)	15.36 ± 10.01	16.77 ± 9.04	26.47 ± 9.63	8.6 ***	c > (b, a) ***
Job Satisfaction (WES-10)	22.29 ± 5.11	21.71 ± 3.36	18.67 ± 7.12	4.5 *	(a, b) ** > c
Perceived Stress Scale	22.42 ± 7.10	24.74 ± 7.01	29.47 ± 7.50	6.0 **	c > b * > a **

* *p* < 0.05; ** *p* < 0.01; *** *p* < 0.001. Group a = No alcohol consumption; Group b = Non-problematic-consumption; Group c = Problematic-alcohol-consumption.

**Table 4:** Odds Ratio for problematic alcohol consumption in male and female professors from higher to lower.

	Criteria	Odds Ratio	Confidence Interval 95%	
			Lower	Upper
**Male Professors**				
Node 7	Job satisfaction > 23 and perceived stress > 32	--	--	--
Node 5	Job satisfaction > 19 and perceived stress > 32	4.9	1.5	16.3
Node 1	Job satisfaction ≤ 19	2.6	1.1	5.8
Node 4	Job satisfaction > 19 and perceived stress > 22 ≤ 32	1.4	0.6	2.9
Node 6	Job satisfaction < 23 and perceived stress > 32	0.7	0.1	5.6
Node 2	Job satisfaction > 19	0.4	0.2	0.1
Node 3	Job satisfaction > 19 and perceived stress ≤ 22	0.1	0,1	0,4
**Female Professors**				
Node 4	Job satisfaction ≤ 22 and psychological inflexibility > 17	21.6	4.5	103.6
Node 1	Job satisfaction ≤ 22	8.7	1.1	69.2
Node 6	Job satisfaction >22 and perceived stress > 34	2.4	0.3	21.8
Node 2	Job satisfaction > 22	1.3	0.1	10.8
Node 3	Job satisfaction ≤ 22 and psychological inflexibility < 17	0.1	0.1	1.2
Node 5	Job satisfaction > 22 and perceived stress < 34	--	--	--
